# Recombinant Expression of ABCC2 Variants Confirms the Importance of Mutations in Extracellular Loop 4 for Cry1F Resistance in Fall Armyworm

**DOI:** 10.3390/toxins14020157

**Published:** 2022-02-21

**Authors:** Laura Franz, Klaus Raming, Ralf Nauen

**Affiliations:** Bayer AG, Crop Science Division, R&D, Pest Control, 40789 Monheim am Rhein, Germany; laura.franz@bayer.com (L.F.); klaus.raming@bayer.com (K.R.)

**Keywords:** *Bacillus thuringiensis*, *Spodoptera frugiperda*, resistance, Cry1, Sf9 cells, ABC transporter

## Abstract

Fall armyworm (FAW), *Spodoptera frugiperda*, is a highly destructive and invasive global noctuid pest. Its control is based on insecticide applications and *Bacillus thuringiensis* (Bt) insecticidal Cry toxins expressed in transgenic crops, such as Cry1F in Bt corn. Continuous selection pressure has resulted in populations that are resistant to Bt corn, particularly in Brazil. FAW resistance to Cry1F was recently shown to be conferred by mutations of ATP-binding cassette transporter C2 (ABCC2), but several mutations, particularly indels in extracellular loop 4 (ECL4), are not yet functionally validated. We addressed this knowledge gap by baculovirus-free insect cell expression of ABCC2 variants (and ABCC3) by electroporation technology and tested their response to Cry1F, Cry1A.105 and Cry1Ab. We employed a SYTOX^TM^ orange cell viability test measuring ABCC2-mediated Bt toxin pore formation. In total, we tested seven different FAW ABCC2 variants mutated in ECL4, two mutants modified in nucleotide binding domain (NBD) 2, including a deletion mutant lacking NBD2, and *S. frugiperda* ABCC3. All tested ECL4 mutations conferred high resistance to Cry1F, but much less to Cry1A.105 and Cry1Ab, whereas mutations in NBD2 hardly affected Bt toxin activity. Our study confirms the importance of indels in ECL4 for Cry1F resistance in *S. frugiperda* ABCC2.

## 1. Introduction

*Spodoptera frugiperda* (J.E. Smith) (Lepidoptera: Noctuidae), fall armyworm (FAW), is a highly-destructive moth pest native to the tropical and subtropical Americas that feeds on a broad range of host plants [1,2], including important row crops such as corn, cotton and soybean [3,4]. FAW recently invaded the Eastern Hemisphere and the first outbreaks were described in West Africa in 2016 [5], from where it rapidly dispersed to many countries in Sub-Saharan Africa [6]. In 2018, FAW was detected in India [7], followed by other countries in the Asia-Pacific, including China and Australia [8,9,10].

FAW pest management strategies largely rely on the use of synthetic insecticides and transgenic crops, e.g., corn or cotton expressing *Bacillus thuringiensis* (Bt) insecticidal proteins such as Cry and Vip toxins [11,12,13]. Corn hybrids expressing Bt toxins remain a cornerstone in FAW control in North and South America [11,14]. Transgenic corn expressing Cry1F—particularly targeting FAW—was commercialized in North America and Brazil in 2003 and 2009, respectively [15,16]. Over the last 20 years, Bt crop technology was rapidly adopted for pest management on a large scale, thus augmenting the resistance risk due to increased cross-crop selection pressure on targeted pests, including FAW [17,18,19,20]. The first cases of FAW resistance compromising the field-efficacy of transgenic corn expressing Cry1F were reported only a few years after its introduction in Puerto Rico [21], but also in Brazil [22] and Argentina [23,24]. Subsequent studies on the genetics of Cry1Fresistance in FAW revealed an autosomal (incompletely) recessive inheritance pattern and, in some cases, cross-resistance issues affecting other Bt toxins such as Cry1A.105, Cry1Ac and Cry1Ab [22,25,26,27], but not Vip3A or the foliar Bt products XenTari and DiPEL [15,28].

After ingestion, Cry toxins are proteolytically activated and interact (most likely sequentially) with potential receptor proteins on the surface of midgut epithelial cells, subsequently facilitating pore formation by membrane insertion of Cry toxin oligomers, leading to feeding cessation, followed by death of the targeted pest [29,30]. The exact molecular mode of action of Cry toxins is still not completely clear, but there is an emerging body of evidence that Cry toxins interact particularly with ATP-binding cassette (ABC) transporters to form pores [31,32,33]. The first evidence for such an interaction was provided by a knockout of the ABC subfamily C2 (*ABCC2*) transporter in *Heliothis virescens*, due to a frameshift mutation conferring Cry1Ac resistance [34]. Subsequent studies confirmed the important role of ABCC2 in Cry1 toxin resistance in other Bt resistant lepidopteran species such as *Plutella xylostella* and *Trichoplusia ni* [35], *Bombyx mori* [36], *Spodoptera exigua* [37], and *Helicoverpa armigera* [38]. Likewise, Cry1F resistance in *S. frugiperda* from Puerto Rico was shown to be linked to a 2 bp insertion (GC) in the *ABCC2* gene, resulting in a premature stop codon and a truncated protein, lacking six transmembrane domains and one nucleotide-binding domain [39,40]. Functional baculovirus-mediated expression in Sf9 cells confirmed that the mutant ABCC2 did not function as a Cry1F receptor, whereas the expression of wildtype ABCC2 renders Sf9 cells sensitive to Cry1F (and a few Cry1A toxins), as measured by cytotoxicity assays [39,40]. CRISPR/Cas9-mediated *ABCC2* knockout in FAW confirmed its importance for Cry1F toxicity in vivo [41]. Flagel et al. [40] additionally detected splice disruption sites on exon 4 of the FAW *ABCC2* gene. However, the mutant *ABCC2* GC-insertion allele, detected at high frequency in Puerto Rican FAW populations, was completely absent in Brazilian samples collected at 15 locations in 2016 and screened using a Taqman allelic discrimination assay [40]. This was later confirmed by monitoring studies using alternative mutant allele genotyping strategies to analyse preserved FAW samples from Puerto Rico and Brazil [42,43].

The molecular mechanism of Cry1F resistance in Brazilian FAW populations was discovered only recently and were shown to be functionally linked to mutations in extracellular loop 4 (ECL4) of *ABCC2*, which result in a GY deletion and a P799K/R amino acid substitution [44]. Another amino acid substitution, G1088D, close to nucleotide binding domain 2 (NBD2) of ABCC2 was also detected in a Cry1F resistant Brazilian FAW strain, but not tested for functional implications on Cry1F binding [44]. The importance of ECL4 (and ECL2) for Cry1F interaction with FAW ABCC2 was supported by functional studies with recombinantly expressed ABCC2 variants in High-5 cells, which differed in their ECL sequences and Cry1F responsiveness [45]. Whole genome sequencing approaches recently confirmed the presence of mutations in ECL4 of *ABCC2* in Brazilian FAW samples [46], but not in invasive populations collected in Eastern Hemisphere countries, including China [47]. When assessing the frequency of the GY deletion in ECL4 of ABCC2 in Brazilian FAW samples by pooled population sequencing, additional indels have been detected in ECL4 which were not yet functionally tested for their impact on Cry1F toxicity [44]. Furthermore, it remains unclear if other toxins than Cry1F are affected by these reported mutations in ECL4 of *S. frugiperda* ABCC2, including GY-deletion and P799K/R.

The objective of our study was to address this knowledge gap by functionally testing the interaction of several recombinantly expressed FAW ABCC2 variants mutated in ECL4 with Cry1F, Cry1A.105 and Cry1Ab. Furthermore, we investigated the impact of G1088D (NBD2) and a mutant ABCC2 completely lacking NBD2 on Cry1F and Cry1Ab mediated pore formation. We achieved this by baculovirus-free insect cell expression of *ABCC2* variants by electroporation technology and employing a highly efficient fluorescence-based cytotoxicity assay based on SYTOX^TM^ orange in 384-well plates.

## 2. Results

### 2.1. Effect of SfABCC2 ECL4 Mutations on Cry1F, Cry1A.105 and Cry1Ab Toxicity

**Figure 1:**
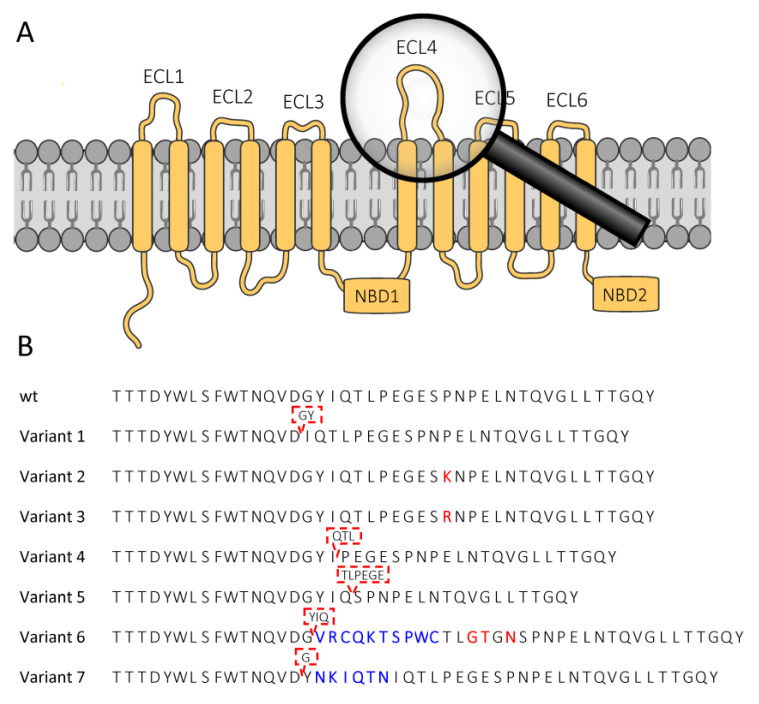
Functionally expressed *Spodoptera frugiperda* ABCC2 transporter variants and mutations in ECL4 (wt. = wildtype). (**A**) Schematic structure of SfABCC2, showing the six extracellular loops (ECLs) and two nucleotide binding domains (NBDs). (**B**) Mutations described in the ABCC2 ECL4 of Cry1F-resistant *S. frugiperda* populations from Brazil [44] tested in this study. Red: amino acid substitution; blue: inserted amino acids; red dashed box: deleted amino acids.

In a previous study [44], we identified several mutations in the ABCC2 ECL4 of Cry1F-resistant FAW populations from Brazil (Figure 1). To investigate the effect of these mutations on the ability of the FAW ABCC2 (SfABCC2) to serve as a Bt toxin receptor, SfABCC2 wildtype and variant proteins were functionally expressed in Sf9 cells transfected by electroporation technology, rather than commonly-used baculoviruses.

The toxicity of the trypsin-activated Bt toxins Cry1F, Cry1A.105 and Cry1Ab to these ABCC2-expressing Sf9 cells was determined in a SYTOX^TM^ orange cytotoxicity assay measuring the membrane permeabilization upon incubation with Bt toxins (Table 1). The expression of the SfABCC2 wildtype protein in Sf9 cells conferred susceptibility to Cry1F, Cry1A.105 and Cry1Ab with EC_50_-values of 0.395, 0.0946 and 0.110 nM, respectively, while non-transfected Sf9 cells were not affected. Sf9 cells expressing the SfABCC2 variants 1 (GY-deletion) and 4–7 (see Table 1) showed no susceptibility to Cry1F at the highest tested concentration of 1000 mM, resulting in Cry1F resistance ratios (RR) of >2532-fold. These variants contain ECL4 indels of various length (see Figure 1B). Similarly, the amino acid substitutions P799K and P799R (variants 2 and 3, respectively) conferred high resistance to Cry1F, i.e., RR 630- and 1643-fold, respectively. In contrast, the toxicity of Cry1Ab and Cry1A.105 to the cells expressing the SfABCC2 ECL4 mutant variants was much less affected compared with Cry1F. For the variants 1–4, in which, at most, three amino acids in the ECL4 were altered (substituted or deleted), low RRs of 3.6- to 16.7-fold were observed. For the variants with larger indels (variants 5–7), RRs of 46.3 to 301.3 were determined. Compared to all other variants, the GY-deletion mutant showed the lowest RRs against Cry1Ab and Cry1A.105, i.e., 4.5- and 3.6-fold, respectively.

### 2.2. SfABCC3 Is a Receptor for Cry1Ab but Not Cry1F

Next, we tested the toxicity of Cry1F and Cry1Ab to Sf9 cells expressing SfABCC3. Expression of the SfABCC3 conferred high susceptibility of the Sf9 cells to Cry1Ab (EC_50_ 3.32 nM), whereas no toxicity of Cry1F to SfABCC3-expressing Sf9 cells was observed at the highest tested concentration of 1000 nM (Table 1), suggesting that the ABCC3 shows a functional redundancy to ABCC2 as a Cry1Ab receptor but does not function as a Cry1F receptor, mediating pore formation.

### 2.3. ABCC2 Gating Activity has no Effect on Cry1 Activity

**Figure 2:**
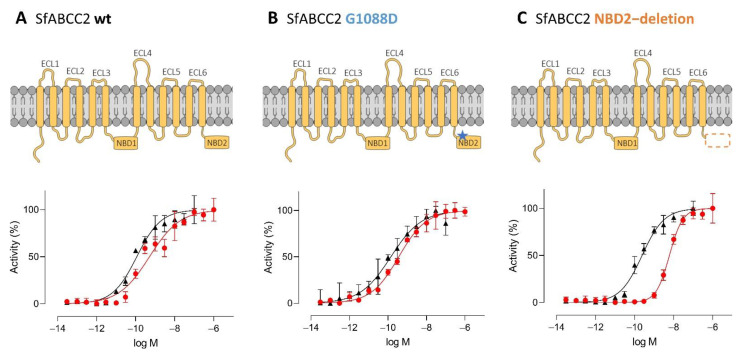
Effect of mutations in the SfABCC2 nucleotide binding domain 2 (NBD2) on the activity of Cry1F and Cry1Ab. Sf9 cells expressing SfABCC2 wild-type (wt.) (**A**), SfABCC2 G1088D [GenBank OM169178] (**B**) or SfABCC2 NBD2-deletion [GenBank OM169179] (**C**) (see schematics) were treated with activated Cry1F and Cry1Ab, respectively, and the toxicity was determined in a SYTOX^TM^ orange cytotoxicity assay measuring membrane permeabilization. Data are mean values ± CI95% (*n* = 4). Blue star: G1088D substitution; orange dashed box: deleted NBD2.

In addition to the mutations in the ECL4, we previously observed an amino acid substitution (G1088D) close to the ABCC2 NBD2 of Cry1F- and Cry1Ab-resistant FAW strains [44]. We expressed the SfABCC2 G1088D variant as well as a variant in which NBD2 was completely deleted in Sf9 cells to investigate the effect of these mutations and the necessity of the ABCC2 gating activity to function as a Bt toxin receptor (Figure 2). The mutation G1088D had no significant effect on the toxicity of Cry1F (EC_50_ 0.386 nM, CI95% 0.255–0.584; EC_95_ 66.8 nM, CI95% 18.6–240) and Cry1Ab (EC_50_ 0.0806 nM, CI95% 0.0656–0.0992; EC_95_ 2.23 nM, CI95% 1.24–4.02) when compared to wild-type ABCC2 (Figure 2A,B; Table 1). Additionally, the expression of the NBD2-deletion variant conferred nanomolar susceptibility of Sf9 cells to Cry1F and Cry1Ab. The deletion of the NBD2 had no significant effect on the toxicity of Cry1Ab (EC_50_ 0.183 nM, CI95% 0.154–0.218), but resulted in a 15-fold decreased Cry1F toxicity (EC_50_ 5.77 nM, CI 95% 5.26–6.34) compared to the wild-type ABCC2 (Figure 2C; Table 1). However, based on EC_95_ values there was no significant difference in Cry1F efficacy between wild-type ABCC2 (47.0 nM, CI95% 25.7–85.8) and the NBD2-deletion mutant (45.9 nM, CI95% 35.4–59.5).

## 3. Discussion

There is an emerging body of evidence that ABC-transporters are critical determinants of three-domain (3D) Cry toxin-mediated toxicity and resistance in several lepidopteran species [29,32,33,49,50,51]. This is also supported by results obtained with ABCC2 knockout lines of *S. frugiperda* expressing high levels of resistance against Cry1F and Cry1Ab [41]. Studies with genome-edited *S. exigua* confirmed a crucial role for ABCC2, but not ABCC3 in Cry1F and Cry1Ac toxicity [50]. In *B. mori* only the double knockout of ABCC2 and ABCC3 conferred Cry1F resistance [51], however, in contrast, the double knockout in FAW was lethal [49].

Recently, a number of studies have linked mutations in the *ABCC2* gene to Cry1F resistance in FAW populations [39,40,44]. High levels of field-relevant Cry1F resistance in FAW have been shown to be caused by truncated non-functional ABCC2 receptors in strains from Puerto Rico [39,40], whereas a GY-deletion and P799K in ECL4 has been shown to result in Cry1F resistance in Brazilian FAW populations [44]. This result is supported by another study systematically investigating the crucial role of ECL4 and other ECLs for Cry1F toxicity [45]. However, recently, a whole genome sequencing approach identified a novel mutation in *ABCC2* in two Brazilian FAW individuals, which were heterozygous for a 12 bp insertion, leading to a premature stop codon [47].

The data obtained in the present study confirmed the key role of FAW ABCC2 and ABCC3 as Cry1Ab receptors, but did not suggest functional redundancy of ABCC2 and ABCC3 as Cry1F receptors, as for example shown for Cry1F in *B. mori* [51], or for Cry1A toxins in *H. armigera* [52]. We observed no membrane permeabilization in Sf9 cells expressing ABCC3 when incubated with Cry1F, whereas Cry1Ab and Cry1A.105 interacted with both ABCC2 and ABCC3 at low nanomolar concentrations.

Cry toxin induced cytotoxicity is often quantified in cell-based assays by the measurement of lactate dehydrogenase (LDH) release indicating necrotic cell death [53]. This is a commonly-used assay to measure membrane permeabilization, along with the microscopic assessment of cell swelling mediated by water influx via aquaporins [53,54,55]. We employed a slightly different approach to determine the effect of ABCC2 mutations on Cry toxin-facilitated pore formation by using SYTOX^TM^ orange, a highly sensitive fluorescent DNA stain, allowing us to quantitatively measure Sf9 cell membrane permeabilization across a wide range of toxin concentrations in 384-well plates.

Our work supplements findings made in a previous study, where pooled population sequencing identified field-derived allelic variations of FAW ABCC2, particularly indels in ECL4, most likely arisen independently as soft selective sweeps [44]. By functional expression of mutant FAW ABCC2 receptors in Sf9 cells, we confirmed the important role of ECL4 for Cry1F interaction and resistance.

A GY-deletion was previously detected at high frequency and was shown to be linked to high levels of Cry1F resistance in a resistant Brazilian field-collected population, Sf_Des [44]. Two more mutations in ABCC2, P799K/R and G1088D, were found in Sf_Des and another Cry1F resistant strain, Sf_Cor [44]. The impact of GY-deletion and P799K on ABCC2-mediated pore formation by Cry1F was confirmed by the functional expression of mutant receptors in Sf9 cells and subsequent cytotoxicity measurements after Cry1F treatment [44], whereas P799R (detected in 14 Brazilian populations), in addition to other ECL4 indels and G1088D (close to NBD2), remained untested. Here, we provided substantial evidence that those ECL4 mutations in FAW ABCC2 not yet tested are highly relevant, resulting in Cry1F resistance levels between 600- and >2500-fold in cellular assays, whereas G1088D (close to NBD2) did not affect Cry1F-induced pore formation in Sf9 cells. In fact, we demonstrated that the deletion of the entire NBD2 domain did not substantially alter the activity of Cry1F and Cry1Ab, a finding in line with earlier results showing that *B. mori* ABCC2 devoid of NBD2 retained its Cry1Aa receptor activity [56]. In addition, these authors demonstrated, by investigating 29 ABCC2 mutants, that a conserved “DYWL” motif in ECL4 is a determinant for Cry1Aa binding. In a more recent study, *S. frugiperda* and *Mythimna separata* ABCC2 ECL4 was divided into three domains, M1-M3, which were shown to differentially contribute to Cry1F selectivity [45]. The authors replaced the different ECL’s between species and demonstrated that *M. separata* ABCC2 expressing the entire coding sequence of FAW ECL4 showed a remarkable increase in Cry1F sensitivity compared to *M. separata* wildtype ABCC2 [45]. All allelic ABCC2 variants tested here have indels downstream from the conserved “DYWL” motif (Figure 1), particularly at the interface of the M1 (“TTTDYWLSFWTNQVDGYIQTL”) and M2 (“PEGESPNPELDT”) regions, supporting the importance for Cry1F interaction of these sequence stretches, as defined by Liu et al. [45]. We also noticed a single polymorphism at position 804 (in M2 of ECL4) where our FAW wildtype ABCC2 sequence [GenBank OL955491] harbours an asparagine in contrast to aspartic acid, reported by Liu et al. [45].

Interestingly, neither the GY-deletion nor the P799K/R amino acid substitution showed a major effect on Cry1Ab and Cry1A.105 toxicity towards Sf9 cells expressing these ABCC2 variants when compared to wildtype ABCC2. Cry toxin proteins have three principle domains (DI-III) and the critical role of domain II for *B. mori* ABCC2 receptor binding has been recently shown for Cry1Aa [57]. Domain II of Cry1Ab and chimeric Cry1A.105 is identical, but different from Cry1F [48], possibly explaining the observed difference in specificity towards functionally expressed FAW ABCC2 variants mutated in ECL4. Indeed, the EC_50_-values measured for Cry1Ab and Cry1A.105 on different ABCC2 variants significantly coincided, supporting the role of domain II for ABCC2 interaction and/or binding. Additional studies are warranted to identify and better understand the structural determinants of Cry1F selectivity in comparison to other Cry1 toxins, e.g., by mutagenesis of domain II loop regions, resulting in disabled proteins lacking functional features essential for toxicity, such as recently demonstrated with Cry proteins modified in domain I involved in pore formation [58].

Our results indicated that the >400-fold Cry1Ab resistance described in the Brazilian strain Sf_Des [59] is not linked to the presence of GY-deletion and P799K in ABCC2 ECL4. Further research is necessary to investigate if other extracellular loop structures such as ECL2 (described for *B. mori* ABCC2 [53]), or alternative receptors contribute to Cry1Ab resistance in FAW; except cadherin which was shown to be not involved in Cry1F and Cry1Ab susceptibility in FAW [60]. Indeed, it has been demonstrated that extracellular loops other than ECL4 contribute to Cry1 toxin specificity, such as ECL1 to Cry1Ac, binding to different *Spodoptera* ssp. ABCC2 transporters [61]. Here, Cry1Ab and Cry1A.105 were much less affected than Cry1F by the investigated indels in ECL4 and still induced pore formation at nanomolar concentrations in Sf9 cells, irrespective of the ABCC2 mutant variant expressed. It is currently unknown if the low-resistance ratios in vitro for Cry1A.105 and Cry1Ab would translate into significant resistance in vivo. Further work is necessary to explore the molecular determinants of Cry1A toxin specificity to FAW ABCC2 and to shed light on potential cross resistance issues between Cry1F and other Cry1 toxins, including Cry1Ab and Cry1A.105.

## 4. Conclusions

Our study, employing a baculovirus-free expression system of ABCC2 mutant receptors in Sf9 cells, combined with a SYTOX^TM^ orange cell viability stain, underpinned the importance of ABCC2, particularly ECL4, for Cry1F mediated pore formation and toxicity in FAW. The failure of Cry1F to facilitate cytotoxicity in Sf9 cells expressing FAW ABCC3 suggests the absence of functional redundancy between ABCC2 and ABCC3, which contrasts to the results obtained for Cry1Ab and Cry1A.105. Lack of the NBD2 in FAW ABCC2 hardly affected Cry1F and Cry1Ab toxicity, supporting earlier findings with Cry1A toxins and beet armyworm ABCC2 [62], as well as silkworm ABCC2 [56].

## 5. Materials and Methods

### 5.1. Insect Cell Culture

Sf9 cells were maintained in suspension culture at 27 °C, 120 rpm in Sf900 II culture medium supplemented with 1% foetal bovine serum (FBS) (all from Thermo Fisher Scientific, Waltham, MA, USA), hereafter referred to as cell medium. Cells were passaged twice a week at a density of 4 × 10^5^ cells/mL. One day before the transfection, a 4-day culture was split into a density of 1.5 × 10^6^ cells/mL.

### 5.2. Expression of ABC-Transporter Variants in Sf9 Cells

For the functional expression of the ABC-transporter variants, Sf9 cells were transiently transfected by electroporation technology using a MaxCyte STx transfection system (MaxCyte, Gaithersburg, MD, USA). The ABCC2 and ABCC3 sequences with a C-terminal 3xFLAG-tag (GenBank accession numbers are stated in Table 1), cloned into pIB/V5-His vector, were purchased from Thermo Fisher Scientific (Waltham, MA, USA). For the transfection, Sf9 cells were harvested by centrifugation (120× *g*, 5 min, 22 °C) and resuspended in 50:50 electroporation buffer (MaxCyte, Gaithersburg, MD, USA): Sf900 II medium to achieve a density of 1 × 10^8^ cells/mL. Cells were mixed with plasmid DNA (100 µg pDNA (in H_2_O)/mL cell suspension) and transferred into the electroporation processing assemblies. Cells were electroporated using the MaxCyte Sf9 protocol. After the electroporation, the cells were transferred into a 6-well plate, mixed with one volume of Sf900 II medium, as well as DNase I (final concentration 0.05 U/µL, Thermo Fisher Scientific, Waltham, MA, USA) and incubated for 30 min at 27 °C, 85% RH. Afterwards, the cells were resuspended in 10 volumes cell medium, and the cell density was adjusted to 3 × 10^5^ cells/mL. The cells were seeded into black µCLEAR 384-well plates (Greiner Bio-One, Frickenhausen, Germany) at 15,000 cells/well and incubated for 48 h at 27 °C, 85% RH.

### 5.3. SYTOX^TM^ Orange Cytotoxicity Assay

To determine the toxicity of activated Bt toxins to Sf9 cells expressing ABC-transporter variants a SYTOX^TM^ orange stain was employed [63]. SYTOX^TM^ orange is a highly sensitive fluorescent dye that stains DNA in cells with permeabilized membranes. Trypsin-activated and purified proteins were kindly provided by Kristina Berman (Bayer Crop Science, Chesterfield, MO, USA) and were prepared as previously described [48]. For the cytotoxicity assay, the medium was removed from the cells and replaced with a 25 µL buffer (20 mM HEPES, 5 mM NaHCO_3_, 130 mM NaCl, 5 mM KCl, 2 mM CaCl_2_, 1 mM MgCl_2_, pH 7.4 at room temperature) containing activated Bt toxins Cry1F, Cry1Ab or Cry1A.105 in the range of 0.000003 to 1000 nM (Cry1F) and 100 nM (Cry1Ab and Cry1A.105), respectively (four replicates per concentration). The buffer was used as the negative control and 1:10 lysis buffer G1821 (Promega, Madison, WI, USA) as the positive control. After 10 min, 25 µL 4 µM SYTOX^TM^ orange dye (Thermo Fisher Scientific, Waltham, MA, USA) in buffer was added and the plates were incubated 4 h at 27 °C, 85% RH, before fluorescence intensities were measured in the FLIPR Tetra instrument (Molecular Devices, San Jose, CA, USA). EC_50_ values and statistic parameters were calculated using a four-parameter non-linear regression model using GraphPad Prism v8.0.2 (GraphPad Software Inc., San Diego, CA, USA). Data shown are mean values (*n* = 4) ± CI95%. For visualization, the data in Figure 2 were normalized.

## Figures and Tables

**Table 1:** Toxicity (EC_50_-values) of activated Bt toxins (Cry1F, Cry1Ab, Cry1A.105) to Sf9 cells expressing SfABCC2 wt, SfABCC2 variants or SfABCC3 wt. GenBank accession numbers are stated in square brackets.

ABC-Transporter Variant	Cry Protein	EC_50_ (nM) ^a^	95% CI ^b^	Slope (±SE)	RR ^c^
SfABCC2 wt. [GenBank OL955491]	Cry1F	0.395	0.324–0.482	0.616 (0.035)	-
	Cry1Ab	0.110	0.0746–0.163	0.638 (0.073)	-
	Cry1A.105	0.0946	0.0700–0.128	0.492 (0.036)	-
SfABCC2 Variant 1 (GY-deletion) [GenBank OL955490]	Cry1F	>1000	-	-	>2532
	Cry1Ab	0.497	0.379–0.652	0.624 (0.049)	4.5
	Cry1A.105	0.340	0.242–0.476	0.632 (0.062)	3.6
SfABCC2 Variant 2 (P799K) [GenBank OL955488]	Cry1F	249	192–324	1.17 (0.10)	630
	Cry1Ab	0.980	0.845–1.14	0.828 (0.045)	8.9
	Cry1A.105	1.03	0.868–1.21	0.844 (0.053)	10.9
SfABCC2 Variant 3 (P799R) [GenBank OL955487]	Cry1F	649	269–1569	0.922 (0.118)	1643
	Cry1Ab	1.32	1.12–1.56	0.828 (0.051)	12.0
	Cry1A.105	1.58	1.38–1.80	0.789 (0.036)	16.7
SfABCC2 Variant 4 (QTL-deletion) [GenBank OL955486]	Cry1F	>1000	-	-	>2532
	Cry1Ab	0.975	0.826–1.15	0.675 (0.034)	8.9
	Cry1A.105	1.48	1.24–1.78	0.735 (0.044)	15.6
SfABCC2 Variant 5 (TLPEGE-deletion) [GenBank OL955485]	Cry1F	>1000	-	-	>2532
	Cry1Ab	5.09	4.21–6.16	0.864 (0.061)	46.3
	Cry1A.105	6.41	5.65–7.28	1.11 (0.067)	67.8
SfABCC2 Variant 6 (long-insert) [GenBank OL955484]	Cry1F	>1000	-	-	>2532
	Cry1Ab	5.47	4.79–6.25	1.00 (0.057)	49.7
	Cry1A.105	6.61	6.03–7.26	1.36 (0.072)	69.9
SfABCC2 Variant 7 (short-insert) [GenBank OL955489]	Cry1F	>1000	-	-	>2532
	Cry1Ab	20.9	17.8–24.4	0.984 (0.063)	190.0
	Cry1A.105	28.5	23.5–34.5	1.06 (0.089)	301.3
SfABCC3 wt. [sequence from [48]]	Cry1F	>1000	-	-	-
	Cry1Ab	3.32	2.96–3.72	1.30 (0.085)	-
	Cry1A.105	<10 ^d^	-	-	-

^a^ EC_50_ = Effective Concentration resulting in 50% cytotoxicity based on the SYTOX orange fluorescence read-out. ^b^ Confidence interval, 95%. ^c^ Resistance ratio (EC_50_ of SfABCC2 variant divided by EC_50_ of SfABCC2 wt.). ^d^ 10 nM was the only concentration tested due to depleted Cry1A.105 stocks and provided > 50% cytotoxicity.

## Data Availability

We will provide all data generated in this study upon request.
